# Appropriateness of Antibiotic Prescribing in Hospitalized Children: A Focus on the Real-World Scenario of the Different Paediatric Subspecialties

**DOI:** 10.3389/fphar.2022.890398

**Published:** 2022-05-26

**Authors:** Chiara Nasso, Alessandro Scarfone, Igor Pirrotta, Michelangelo Rottura, Domenico Antonio Giorgi, Giovanni Pallio, Natasha Irrera, Violetta Squadrito, Francesco Squadrito, Pierangela Irrera, Vincenzo Arcoraci, Domenica Altavilla

**Affiliations:** ^1^ Department of Clinical and Experimental Medicine, University of Messina, Messina, Italy; ^2^ Department of Human Pathology and Evolutive Age “Gaetano Barresi”, University of Messina, Messina, Italy; ^3^ Department of Biomedical and Dental Sciences and Morphological and Functional Imaging, University of Messina, Messina, Italy

**Keywords:** inappropriate antibiotic use, hospitalized children, active pharmacovigilance, cost, real-word scenario

## Abstract

**Background:** Antibiotics are prescribed for children both in hospital and community settings, particularly at preschool age. Italy shows a high rate of inappropriate antibiotic prescriptions which may represent a serious problem in the hospital scenario. Thus, the aim of this study was to investigate appropriateness of antibiotic prescribing in the context of different paediatric subspecialties in a hospital setting.

**Methods:** Antibiotics prescribing was retrospectively analysed in paediatric patients (0–18 years) admitted in the emergency paediatrics, general paediatrics, paediatric nephrology and rheumatology units between January and December 2019. Patients were stratified by age in neonates, infants, toddlers, children and adolescents. Assessments were conducted by trained local assessors and appropriateness was classified as appropriate, inappropriate and not assessable.

**Results:** Empirical antibiotics were mainly prescribed following a diagnosis of respiratory, gastrointestinal and/or urinary infection. A total of 825 antibiotic prescriptions were recorded in the three subspecialties; 462 antibiotic prescriptions (56%) out of 825 were assessed as inappropriate and 55 prescriptions (6.7%) were not assessable. Inappropriateness considerably varied within subspecialties: the risk of inappropriate antibiotic prescribing was higher in emergency paediatrics and general paediatric than in children, according to age. Ceftriaxone and clarithromycin were the most inappropriate prescribed antibiotics in the emergency paediatrics whereas amoxicillin/clavulanic acid represented the most inappropriate antibiotic prescribed in general paediatrics.

**Conclusion:** The present data may be useful in order to reduce inappropriate antibiotic prescribing in the paediatric setting; antibiotic stewardship and clinical improvement programs in hospital paediatric care are strongly recommended.

## Introduction

Antibiotics are frequently prescribed for children both in hospital and community settings (Principi and Esposito, 2016), particularly at preschool age (Clavenna and Bonati, 2009). Antimicrobial stewardship programs (ASPs) are different in children and adults in terms of outcome measurement: antibiotic dosage in children is based on body weight or body surface area, and therefore, the defined daily dose (DDD) as a preferred measurement of antibiotic consumption is not applicable. An alternative for measuring antimicrobial use is therapy days (i.e., per 1000 patient-days), but unfortunately, such data cannot be compared with adult DDDs (Araujo da Silva et al., 2018).

A quantitative and qualitative variability was observed between countries in antibiotic prescriptions, both in hospitalised and outpatient children and in particular increased antibiotic prevalence rates (number of patients treated with at least 1 antibiotic/100 patients) were detected among non-European paediatric patients (43.8%, range 32.2–65.1%) (Versporten et al., 2013). The Antibiotic Resistance and Prescribing in European Children Point Prevalence Survey (ARPEC-PPS) identified variations among hospitalized children; Africa, Australia, Western Europe and Northern Europe showed a high rate of older narrow-spectrum antibiotics, such as benzylpenicillin, sulfamethoxazole/trimethoprim, amoxicillin and gentamicin. On the contrary, children in Eastern Europe, Southern Europe, Asia, North America and Latin America were mainly treated with broad-spectrum antibiotics, especially third generation cephalosporins, cefepime and meropenem. In addition, a reduction of antibiotic treatment was reported in European neonates (22.8%) than non-European ones (39.4%) (Clavenna, 2015). However, Italy is one of the European countries with the higher rate of inappropriate antibiotic prescriptions (Agenzia Italiana del Farmaco, 2019): antimicrobial drugs were prescribed in 37–61% of hospitalized infants and children (Versporten et al., 2016) and the high rate (20–50%) of these prescriptions are unnecessary or inappropriate (Hecker et al., 2003) (Hulscher et al., 2010). Moreover, different studies demonstrated that many children received broad-spectrum antibiotics to treat viral respiratory infection, thus increasing the risk of antibiotic resistance appearance (Hersh et al., 2011) (McCaig et al., 2003). Additionally, some antibiotics are administered to children for a period considerably longer than that needed or with an incorrect total daily dosage (Levy et al., 2012) (Esposito et al., 2001). However, this abuse and misuse of antimicrobials have numerous negative consequences: an increase of adverse drug reaction (ADR) incidence (Cosgrove, 2006) and risk of toxicity (Donà et al., 2020) were observed. As previously mentioned, the high number of inappropriate antibiotic prescriptions contributes to the antimicrobial resistance (AMR) (Hagedoorn et al., 2020); AMR is described as the capacity of a microorganism (bacterium, parasite, virus or fungus) to avoid an antimicrobial effect against itself (World Health Organization, 2021), therefore it is considered a quickly increasing global public health emergency (World Health Organization, 2021) which has to be managed for epidemiological and economic reasons. In fact, AMR might lead to longer hospital stay, increased risk of mortality, health care costs and treatment failures (Maragakis et al., 2008). Thus, the health and economic consequences of antibiotic resistance are severe. Today, drug-resistant infections lead to approximately 700,000 deaths per year globally. This is projected to increase to 10 million by 2050, with associated costs as high as US $100 trillion worldwide. Each year, in the European Union (EU) alone, 25,000 patients die due to infections caused by multiresistant bacteria, costing society approximately €1.5 billion annually. By 2050, expected cumulative losses due to multiresistance will reach 2.9 trillion USD per year (Machowska and Lundborg, 2019). Previous reports also demonstrated that AMR may be related to a growing incidence of *Clostridium difficile* infection (Baur et al., 2017) as well as negative impact of microbiota (Vangay et al., 2015). For these reasons, antimicrobial stewardship (AS) is a key approach to reduce AMR incidence in hospital settings and may point at a rationale and effective use of drugs in children (World Health Organization, 2021). However, no report has previously analysed the appropriateness of antibiotic prescribing in relation to the different paediatric subspecialties in a hospital setting. Thus, the aim of the present study was to investigate this issue in the real word scenario of an Italian hospital characterized by the presence of several paediatric Units.

## Materials and Methods

In a retrospective observational study, antibiotics prescribing was analysed in paediatric patients (0–18 years) admitted to the University Hospital “G. Martino” of Messina, (Sicily, Italy) for the period between January and December 2019. Antibiotics were classified according to the Anatomical Therapeutic Chemical Classification (ATC) and in particular the prescription of drugs in 2nd level ATC = J01 was evaluated. Emergency paediatrics, general paediatrics, paediatric nephrology and rheumatology were involved in this study and its protocol was approved by the Ethics Committee of the A.O.U. “G. Martino” of Messina (project identification code N°283–20 Bis data of approval 11/11/2020) according to the legal requirements concerning observational studies; patient’s consent to participate was not requested for this kind of study. Data were anonymously recorded by clinical doctors and pharmacists during hospitalization, including patient demographic features, indication for antibiotics, route of antibiotic administration and type of antibiotics as norm of clinical practice. All paediatric patients were stratified by age group: neonates (<28 days), infants (28 days to <1 year), toddlers (1year to <3 years), children (3 years to <12 years), adolescents (12 years to <18 years). Data about consumption and cost of antibiotics prescriptions were extracted from the pharmacy administrative database; the used drugs by ATC code, the consumption in unit dose and the cost in euro (€) were extracted for each unit.

### Antibiotic Appropriateness

The appropriateness of antibiotic prescribing was investigated by consulting the patient’s clinical records of the emergency paediatrics, paediatrics, paediatrics nephrology and rheumatology wards. Patients with at least one antibiotic administration were selected. All clinical information (reason for hospitalization, infections, administered drugs) and socio-demographic (age and sex) features of each patient were collected. The appropriateness of antibiotic prescribing was assessed by the World Health Organization (WHO) AWaRe classification guidelines and by the regional guidelines for appropriate antibiotic prescribing in children (Oteri et al., 2013). Assessments were conducted by trained local assessors (pharmacology trainers, specialist of pharmacology and pharmacist) and appropriateness was classified as follows: appropriate (the best therapeutic approach for the treatment of each diagnosis conducted by clinician), inappropriate (antibiotics not recommended for each diagnosis conducted by clinician) and not assessable (antibiotics not present in the guidelines for the use in a specific diagnosis conducted by clinicians).

### Statistical Analysis

The results were expressed as absolute and relative frequencies of the categorical variables, with 95% confidence intervals (CIs) and as medians and interquartile range (Q1—Q3), respectively. The Mann—Whitney *U* test for independent sample and two—tailed Pearson chi—squared test was performed to compare continuous variables and categorical variables. Odds ratios (ORs) with 95% CIs were estimated for each covariate of interest in univariate (Crude OR) and multivariate (adjusted OR) regression models. A *p-value* < 0.05 was assessed as statistically significant. Statistical analysis was carried out using the SPSS version 23.0 (IBM Corp. SPSS Statistics).

## Results

### Characteristics of Patients

Between January and December 2019, 626 patients were hospitalized in the units of emergency paediatrics, general paediatrics, paediatric nephrology and rheumatology (Table 1). Patients were stratified by age as neonates, infants, toddlers, children and adolescents; the unit of emergency paediatrics admitted the highest number of patients (*N* = 325) and mostly were male (57.8%) and children (37.4%).

**TABLE 1:** Demographic characteristics of patients and antibiotic prescriptions.

Demographic	Number of Patients (%) total = 626	Number of Antibiotic Prescriptions (%) total = 825
Sex male	358 (57.8)	453 (54.9)
Age		
neonates (<28 days)	2 (0.3)	2 (0.2)
infants (28 days to <1 year)	170 (27.2)	226 (27.4)
toddlers (1 year to <3 years)	148 (23.6)	199 (24.1)
children (3 years to <12 years)	234 (37.4)	300 (36.4)
adolescents (12 years to <18 years)	72 (11.5)	98 (11.9)
Paediatric Subspecialties		
Emergency Paediatrics	325 (51.9)	414 (50.2)
General Paediatrics	191 (30.5)	273 (33.1)
Paediatric Nephrology and Rheumatology	110 (17.6)	138 (16.7)

### Antibiotic Prescribing

**FIGURE 1:**
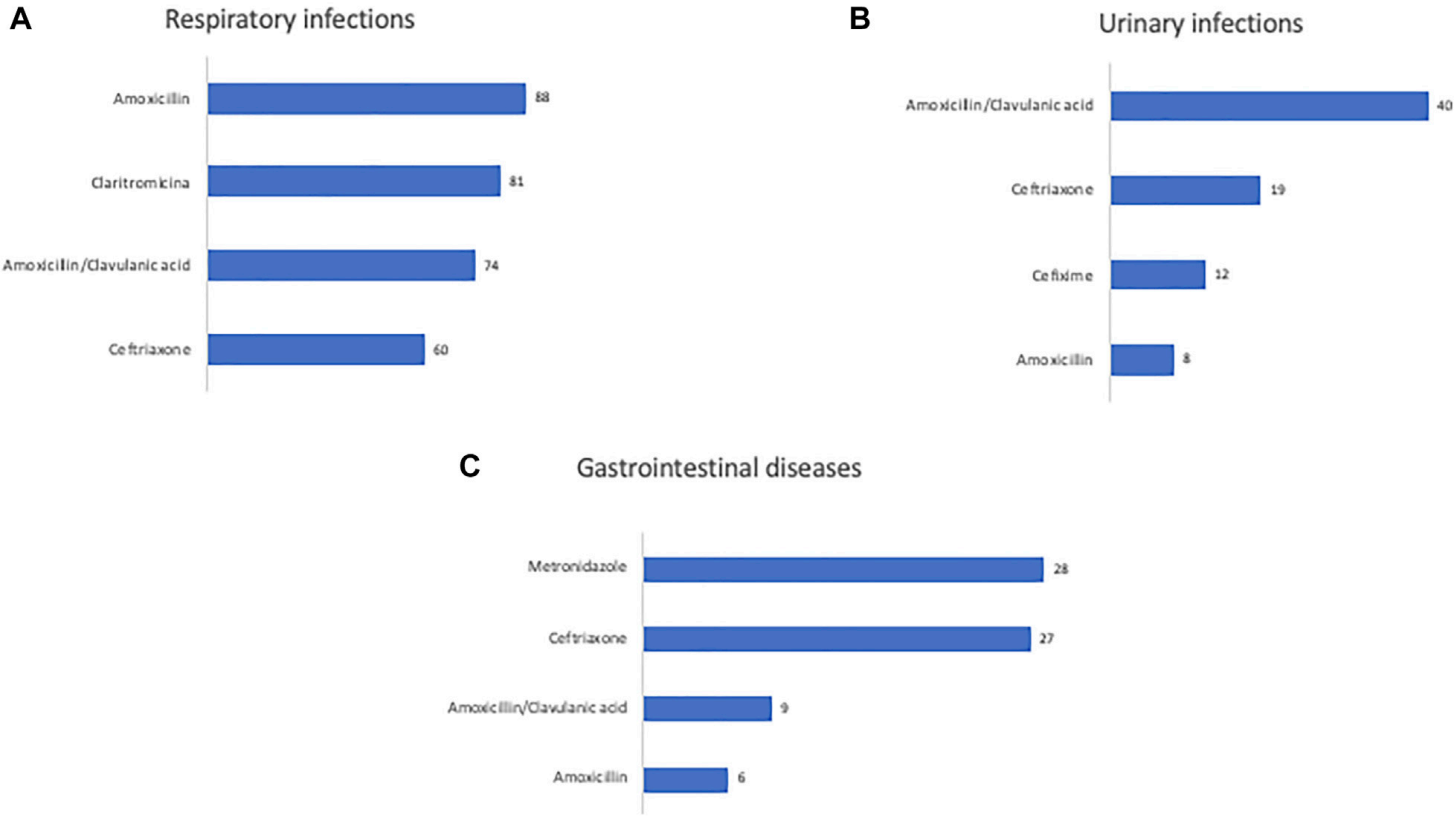
Number of antibiotics prescriptions used for the main diagnoses of patients admitted to pediatric units.

During the hospital stay, 825 prescriptions of antibiotics were recorded (Table 1); more specifically, children received more antibiotics (36.4%) followed by infants (27.4%), toddlers (24.1%), adolescents (11.9%) and neonates (0.2%). The unit of emergency paediatrics showed a greater number of antibiotic prescriptions than the other paediatric subspecialties (Table 1). All the antibiotics were empirically prescribed based on the clinical diagnosis (dermatological diseases, respiratory infections, abscesses, gastrointestinal diseases, central nervous system infection, urinary infections, traumatic wounds). The main hospitalization diagnoses in the paediatrics units were respiratory infections 318 (50.9%), urinary infections 88 (14.0%) and gastrointestinal diseases 83 (13.2%). In addition, among patients with respiratory diseases, 54 (8.6%) patients had upper respiratory tract infections. Figure 1 reports the main prescribed antibiotics in relation to the hospitalization diagnosis. Amoxicillin was the most widely prescribed antibiotic for respiratory infections treatment followed by clarithromycin, amoxicillin/clavulanic acid and ceftriaxone (Figure 1A). Amoxicillin/clavulanic acid was the main antibiotic prescribed for urinary infections treatment, followed by ceftriaxone, cefixime and amoxicillin (Figure 1B). Metronidazole was mostly prescribed for gastrointestinal diseases (Figure 1C) followed by ceftriaxone, amoxicillin/clavulanic acid and amoxicillin.

### Appropriateness

**FIGURE 2:**
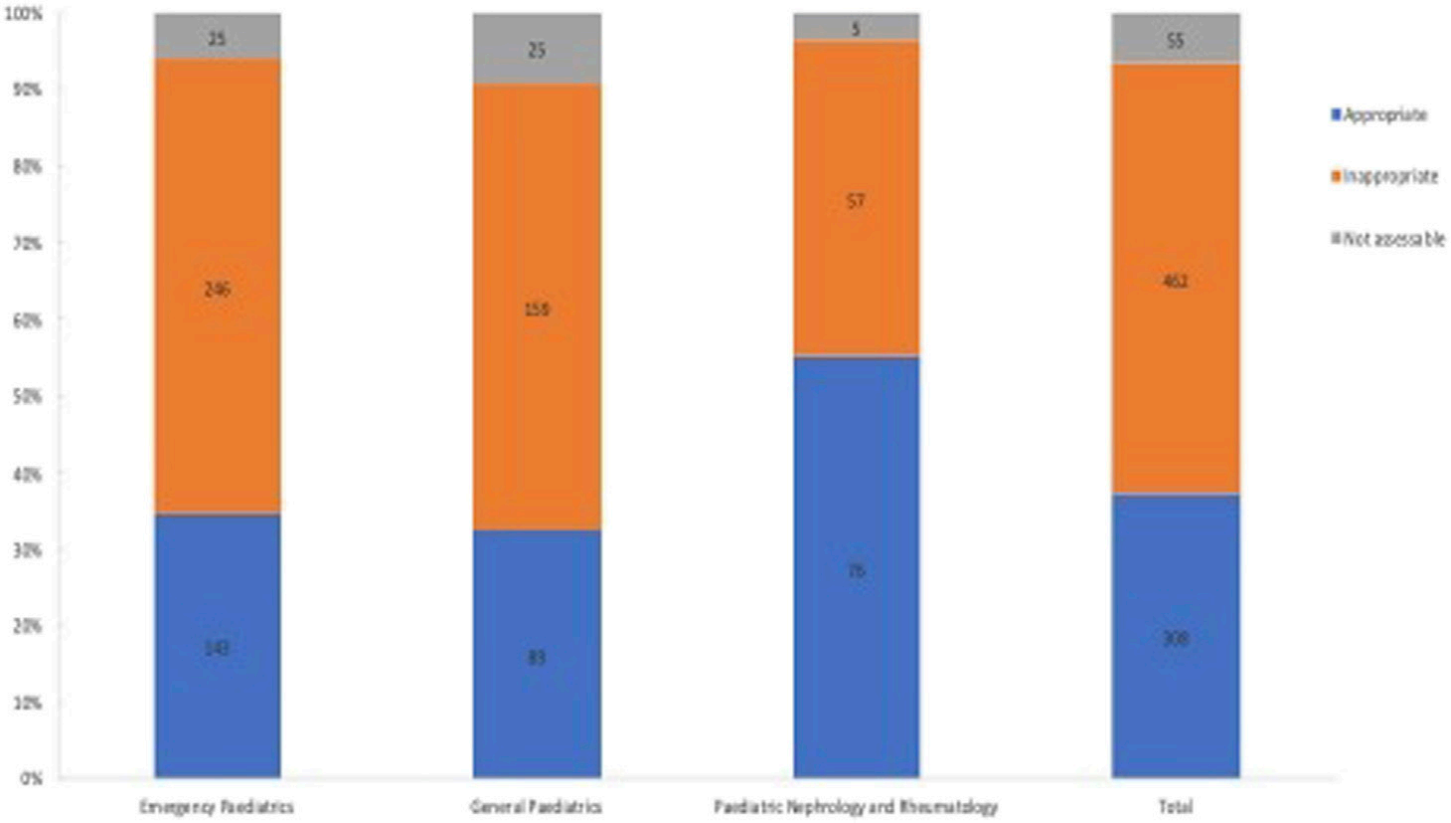
Distribution of appropriateness stratified by paediatric subspecialties.

**FIGURE 3:**
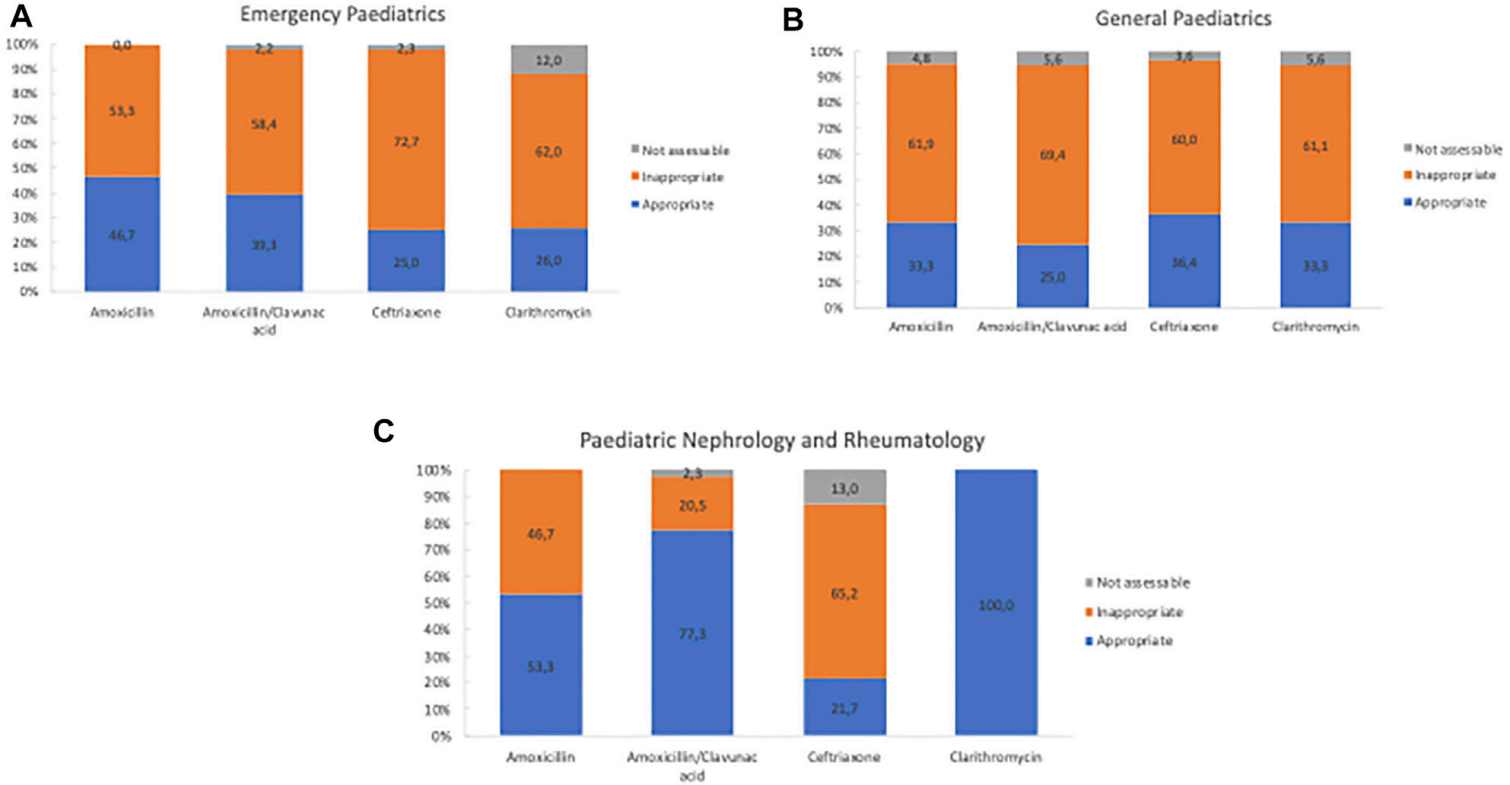
Distribution of appropriateness of the main antibiotic prescriptions stratified by subspecialties.

The appropriateness of antibiotic prescriptions was evaluated by the means of the WHO AWaRe classification and by using the regional guidelines for the antibiotic treatment in the paediatric population. A total of 825 antibiotic prescriptions was recorded in the three subspecialties. 462 antibiotic prescriptions (56%) out of 825 were assessed as inappropriate and 55 (6.7%) were not assessable (Figure 2). Inappropriateness also varied considerably by subspecialty. Emergency paediatric showed the greatest number of inappropriate prescriptions (*n* = 246; 53.24%) followed by general paediatrics (*N* = 159; 34.42%) and paediatric nephrology and rheumatology (*N* = 57; 12.34%) (Figure 2). Figure 3 shows the inappropriate use of the single antibiotic molecule stratified in the several paediatric units. Ceftriaxone and clarithromycin were the most inappropriate antibiotics in the emergency paediatrics; ceftriaxone and amoxicillin were the most inappropriate antibiotics in paediatric nephrology and rheumatology units while amoxicillin/clavulanic acid represented the most inappropriate antibiotic prescribed in general paediatrics (Figure 3). A regression analysis was performed in order to explore the risk factors associated with the occurrence of inappropriate antibiotic use. In this analysis, inappropriate antibiotic prescribing was significantly higher in emergency paediatrics and general paediatric. Conversely, gender, age and hospitalization diagnosis did not significantly influence an inappropriate antibiotic prescribing (Table 2).

**TABLE 2:** Risk factors for inappropriate antibiotic prescribing.

	Crude OR (IC 95%)	*p* Value	Adjusted OR (IC 95%)	*p* Value
Gender, (M)	1.10 (0.81–1.45)	0.595	1.09 (0.80–1.47)	0.588
Hospital Units				
Paediatric Nephrology and Rheumatology	Ref	—	Ref	—
Emergency Paediatrics	2.30 (1.54–3.42)	<0.001	1.81 (1.09–3.01)	0.022
General Paediatrics	2.38 (1.55–3.66)	<0.001	1.85 (1.08–3.17)	0.026
Age				
Neonates and infants	Ref	—	Ref	—
Toddlers	0.68 (0.45–1.02)	0.062	0.67 (0.44–1.03)	0.069
Children	0.70 (0.48–1.00)	0.050	0.70 (0.48–1.04)	0.078
Adolescents	0.73 (0.44–1.21)	0.220	0.82 (0.47–1.42)	0.475
Hospitalization diagnoses				
Urinary infections	Ref	—	Ref	—
Gastrointestinal diseases	1.83 (1.02–3.29)	0.042	1.13 (0.56–2.23)	0.728
Respiratory diseases	2.63 (1.66–4.17)	<0.001	1.69 (0.94–3.05)	0.079
Other diagnosis	2.66 (1.64–4.32)	<0.001	1.76 (0.98–3.19)	0.060

### Utilization and Expenditure

In our study, drug utilization and expenditure were also evaluated in the paediatric units throughout the observation period. The 18.8% of the 26.617 consumed drugs in all paediatric units was antibiotics and more than half of the antibiotic utilization (54.9%) was observed in the emergency paediatric unit (Table 3). Moreover, a total of 53.318,06 euros was spent for drugs in all paediatrics units and the highest cost (60,7%) was related to general paediatrics unit with an amount of 32354,11 euros. When only the expenditure for antibiotics (2nd level ATC = J01) was considered the total cost was 8.003,06 euros and emergency paediatrics unit showed the greatest cost (61,9%) with an amount of 4956,36 euros.

**TABLE 3:** Utilization and expenditure of the total amount of drugs and antibiotics (2nd level ATC = J01) for each single paediatrics units.

	Utilization (unit Dose)		Cost (€)	
Paediatrics units	Total N = 26.617 (100%)	J01 N = 4.996 (100%)	Total € 53.318,06 (100%)	J01 € 8.003,06 (100%)
Emergency Paediatrics	14702 (55,2%)	2743 (54,9%)	14897,44 (27,9%)	4956,36 (61,9%)
General Paediatrics	6435,88 (24,2%)	1752 (35,1%)	32354,11 (60,7%)	2374 (29,7%)
Paediatric Nephrology and Rheumatology	5480 (20,6%)	501 (10,0%)	6066,51 (11,4%)	336,35 (4,2%)

## Discussion

This retrospective study provides an analysis of appropriateness of antibiotic prescribing in hospitalized paediatric patients allocated in different subspecialties in agreement with their medical needs. As far as we know, no previous study has focused attention on the antibiotic prescribing related to the different paediatric subspecialties. This issue was investigated taking advantage of the availability of a real word scenario: a hospital characterized by the presence of several paediatric units. Our results showed the occurrence of a high rate of antibiotic inappropriate prescription; more than half of the prescribed antibiotics was inappropriate. Therefore, the co-presence of several paediatric subspecialties units does not allow a better management of antibiotic prescribing, even if they are empirically prescribed based on the clinical diagnosis. Furthermore, we tried to investigate the “clinical scenario” more “at risk” of antibiotic inappropriateness. As expected, the emergency paediatrics unit showed the higher rate of inappropriate antibiotic prescribing. This is not surprisingly if the “characteristics of emergency” of this clinical setting are taking into account. Indeed, previous data have suggested that antibiotics represent the most commonly drugs prescribed with inappropriateness in the general emergency department either in adults and children, in turn causing unwanted adverse events and contributing to the development of therapeutic failure and antimicrobial resistance (Denny et al., 2019; Denny et al., 2020). However, the same disaggregated information for emergency department exclusively dedicated to the paediatric population is scarce. The present data confirm that the availability of “a paediatric emergency unit” does not cause any improvement in the appropriateness of antibiotic therapy and in the selection of the right antibiotic, likely as a consequence of the need to treat patients for a fast clinical evaluation without any in-depth further analysis of the clinical status. Furthermore, our results showed that ceftriaxone and clarithromycin were the most inappropriate molecules. By contrast, the paediatric nephrology and rheumatology units showed a tendency towards a more rationale and appropriate use of the antibiotics. The reason of this finding may be ascribed to the availability of a closer clinical evaluation of patients admitted to these yards that allows a better understanding of the underling infectious disease. Paediatric population was also stratified by age, but any significant correlation was observed between age and inappropriate antibiotic prescribing. This result is, at least in part, in disagreement with a large Australian nationwide survey assessing over 6000 prescriptions for 4000 hospitalized paediatric patients (McMullan et al., 2020) In fact, this Australian survey showed that older age was significantly associated with inappropriate prescribing (McMullan et al., 2020). Indeed, information on the antibiotic prescribing in hospitalized paediatric population is scarce in Italy and the most relevant data are available for primary care patients: more specifically, it has been shown that during 2019, in Italy, 40.9% of children under the age of 13 received at least one antibiotic prescription, with an average of 2.6 prescriptions for child and with a high rate of inappropriateness (Agenzia Italiana del Farmaco, 2019). Our results confirm, in a paediatric hospital setting, the poor attitude to an appropriate antibiotic prescribing. As already known, paediatric population shows a high frequency of exposure to antibiotics (Piovani et al., 2013). For this reason, an adequate correlation of the qualitative-quantitative profile of antibiotic administration could limit the risk of adverse events appearance and reduce the paediatric expenditure for drugs of the National Health System (NHS). According to a recent study by the ARPEC (Antibiotic Resistance and Prescribing in European Children project group) carried out on hospitalized paediatric patients, ceftriaxone was the active ingredient with the highest prevalence of use (9.8%), followed by amoxicillin and clavulanic acid (7.6%) (Versporten et al., 2016). In our study amoxicillin was the most widely used antibiotic drug (44.4%) for the respiratory infection treatment followed by clarithromycin, amoxicillin/clavulanic acid and ceftriaxone. These results were in accordance with the regional guidelines for the antibiotic treatment in the paediatric population that indicates amoxicillin use for all the respiratory infections (Oteri et al., 2013). By contrast, amoxicillin/clavulanic acid was the main antibiotic prescribed for the urinary infections management, followed by ceftriaxone, cefixime and amoxicillin. Indeed, the different pattern of antibiotic prescription may be characteristic of the hospital setting because of polytherapy of hospitalized patients. Furthermore, drug use and expenditure were also explored in the several paediatric subspecialties throughout the study. The 18.8% of the 26.617 consumed drugs in all paediatric units was antibiotics and more than half of the antibiotic use (54.9%) was observed in the emergency paediatric unit. This leads us to speculate that an inappropriate antibiotic prescription leads to an exaggerated cost which represents a burden for the national health system as confirmed by the results of Agenzia Italiana del Farmaco 2019 report which showed that antibiotic use represents the 3.6% of Italian public health expenditure with an amount of 692.000.000 Euros (Agenzia Italiana del Farmaco, 2019).

## Limitation

The present study has some limitations. Firstly, this is a single-centre study; secondly, enrolled patients were only hospitalized, and our survey does not capture data on community or primary care, which represent the bulk of antibiotic prescribing. Moreover, antibiogram was not considered during the assessment of the suitability of antibiotics. Also, we did not analyse precise diagnoses in accordance with International Statistical Classification of Diseases, Injuries and Causes of Death (ICD) but only groups of diagnosis.

## Conclusion

Our study suggests the need to implement measures of a close monitoring of antibiotic prescriptions in the paediatric population. Furthermore, our results on antibiotic prescribing in the different paediatric units can be useful for future interventions directed towards reducing inappropriate antibiotic use. More specifically, we have clearly shown that emergency paediatrics unit deserves a close monitoring and additional attention. We suggest that antibiotic stewardship and clinical improvement programs in hospital paediatric care should be strongly recommended. Finally, our study confirms the need to create professional’s positions of clinical pharmacologists with the aim to cooperate and synergize with paediatric clinicians to reduce the exposure of hospitalized patients to an inappropriate antibiotic treatment.

## Data Availability

The dataset generated for this study will not be made publicly available. Further inquires can be directed to the author FS, fsquadrito@unime.it.
